# A Cyclodextrin Polymer for the Removal of Pharmaceuticals as Environmental Pollutants from Water, as Illustrated by the Example of Methylene Blue as a Model Compound

**DOI:** 10.3390/ma18173980

**Published:** 2025-08-25

**Authors:** Iwona Zawierucha, Jakub Lagiewka, Paulina Kapusniak, Damian Kulawik, Sandra Zarska, Tomasz Girek, Aleksandra Ciesielska, Malgorzata Girek-Bak, Wojciech Ciesielski

**Affiliations:** 1 Institute of Chemistry, Jan Dlugosz University in Czestochowa, Armii Krajowej 13/15, 42-200 Czestochowa, Poland; jakub.lagiewka@doktorant.ujd.edu.pl (J.L.); paulina.kapusniak@doktorant.ujd.edu.pl (P.K.); d.kulawik@ujd.edu.pl (D.K.); s.zarska@ujd.edu.pl (S.Z.); t.girek@ujd.edu.pl (T.G.); m.girek-bak@ujd.edu.pl (M.G.-B.); wc@ujd.edu.pl (W.C.); 2 Department of Bioinorganic Chemistry, Faculty of Chemistry, University of Gdańsk, Wita Stwosza 63, 80-308 Gdansk, Poland; olaciesielska66@gmail.com

**Keywords:** cyclodextrin polymer, pharmaceuticals, environmental pollutants, methylene blue, wastewater treatment

## Abstract

This study developed a beta-cyclodextrin polymer crosslinked with citric acid (CDCAPol) for removing water contaminants using methylene blue (MB) as a model compound. The polymer, which features free carboxyl groups and cyclodextrin cavities, demonstrated high adsorptive capacity. Under optimal conditions (0.01 g adsorbent, pH 6, and 50 mg/dm^3^ MB), a removal efficiency of 99.2% was achieved, with a maximum adsorption capacity of 126.58 mg/g as determined by the Langmuir isotherm. Kinetic data fit the best to the pseudo-second-order model, highlighting strong interactions between MB and the polymer. This promising material may find applications in wastewater treatment and environmental protection.

## 1. Introduction

Environmental pollution caused by pharmaceutical residues is an increasingly serious global concern. In recent years, the production and consumption of pharmaceuticals, dietary supplements, and personal protective equipment containing biologically active substances have remained at a high level and continue to grow [1,2]. These substances, including drug metabolites, can enter municipal wastewater and ultimately aquatic environments, where they pose risks to ecosystems and to human and animal health [3,4,5,6]. The presence of pharmaceuticals in surface waters, especially at elevated concentrations, is associated with adverse effects such as genetic mutations, endocrine disruption, and the development of antibiotic-resistant bacterial strains [3,4,5,6,7,8].

Conventional wastewater treatment plants are not fully effective in removing these compounds. As a result, pharmaceutical residues and their transformation products persist in treated effluents, contributing to the contamination of surface waters and, in some cases, even drinking water sources [9,10]. The accumulation of pharmaceuticals in aquatic organisms and their potential biomagnification pose additional ecological and toxicological risks. Chronic exposure, even to low concentrations of certain pharmaceuticals, can lead to developmental abnormalities, allergic reactions, and reproductive disorders in wildlife [7,8].

The effectiveness of pharmaceutical removal depends on the compound’s chemical structure, its biological activity, and the treatment method applied. Advanced water treatment technologies such as membrane bioreactors (MBRs), advanced oxidation processes (AOPs), electrochemical oxidation, photocatalysis, and adsorption are currently under investigation [11]. AOPs, which rely on hydroxyl radicals, are particularly effective in degrading persistent pharmaceutical compounds, including antibiotics and hormones [12,13]. MBRs integrate activated sludge processes with membrane filtration, enabling high removal efficiencies (>95%) while reducing the spatial footprint of treatment systems [14]. Electrochemical AOPs generate reactive species in situ and can achieve removal rates up to 99.9%, with shorter treatment times compared to traditional methods. Adsorption with activated carbon is effective for certain compounds but limited by adsorbent regeneration and disposal issues [15]. Photocatalysis, often involving TiO_2_, enables the mineralization of contaminants into more biodegradable forms, though its efficiency depends on water matrix composition and operational conditions [16].

Pharmaceutical residues found in aquatic systems have emerged as a serious global issue due to their persistence, toxicity, and limited removal through conventional wastewater treatment processes. To address this, a variety of advanced materials have been investigated for their ability to adsorb or degrade pharmaceuticals. Among the most widely used are activated carbon and biochar—porous materials that remove pharmaceuticals such as diclofenac and ibuprofen via hydrophobic and π–π interactions. Biochar, derived from biomass, offers a more sustainable and cost-effective alternative to conventional activated carbon [17,18]. Cyclodextrin-based polymers, particularly those incorporating β-cyclodextrin (β-CD), have gained popularity due to their unique ability to form inclusion complexes with hydrophobic drug molecules. Crosslinking β-CD with citric acid or other agents produces insoluble polymers capable of removing drugs like hormones and anti-inflammatories with high efficiency [19,20]. Metal–organic frameworks (MOFs), such as MIL-101 and ZIF-8, offer extremely high surface areas and customizable pore structures, allowing them to serve as effective adsorbents for diverse pharmaceutical compounds [21]. Magnetic nanoparticles functionalized with specific chemical groups enable the efficient removal of antibiotics like tetracycline and ciprofloxacin while allowing for easy magnetic separation and regeneration [22]. Molecularly imprinted polymers (MIPs) provide high selectivity for specific drugs, such as caffeine, paracetamol, and atenolol, by mimicking molecular recognition sites [23]. In parallel, photocatalytic materials like titanium dioxide (TiO_2_) have been employed to degrade persistent pharmaceuticals via the generation of reactive oxygen species under UV light, offering a pathway for the mineralization of contaminants like steroid hormones [16].

A variety of materials—ranging from natural adsorbents to synthetic nanocomposites—are being developed for pharmaceutical removal from water. Among these, adsorption-based materials remain the most practical approach due to their simplicity and scalability. Cyclodextrin-based sorbents have gained prominence due to their high selectivity, reusability, and ability to simultaneously remove multiple contaminants [24,25]. These materials form stable inclusion complexes with pharmaceutical molecules and can be easily recovered from aqueous solutions.

The aim of this study was to develop and characterize a novel β-cyclodextrin-based polymer crosslinked with citric acid (CDCAPol) to effectively eliminate pharmaceutical contaminants from water. Methylene blue (MB) was selected as a model compound due to its structural similarity to many pharmaceutical substances and its relevance in adsorption studies. This study focused on optimizing the adsorption process and evaluating the polymer’s performance under different physicochemical conditions to assess its potential application in water purification.

## 2. Materials and Methods

### 2.1. Materials

Methylene blue, also known as 3,7-bis(Dimethylamino)phenazathionium chloride (C_16_H_18_ClN_3_S, 319.85 g/mol); β-cyclodextrin (β-CD) (C_42_H_70_O_35_, 1134.98 g/mol); citric acid (CA); and di-Sodium Hydrogen Phosphate were purchased from Sigma-Aldrich, St. Louis, MO, USA. Sodium hydroxide and hydrochloric acid were purchased from Chempur, Piekary Slaskie, Poland. Water was purchased from Hydrolab Ultra Pure Water HLP 10UV system, Hydrolab, Straszyn, Poland.

### 2.2. Synthesis of β-Cyclodextrin Polymer Crosslinked by Citric Acid (CDCAPol)

β-cyclodextrin (1 g), citric acid (3 g), and di-Sodium Hydrogen Phosphate (2 g) were added to a 100 mL round-bottom flask, and the mixture was heated in an oil bath using a magnetic stirrer at 140 °C for 2 h [20]. After cooling to ambient temperature, the crude product was purified through three successive washes with 250 mL of water, followed by suction filtration and drying at 60 °C. The resulting CDCAPol polymer was stored at room temperature in a desiccator.

### 2.3. Analyses

#### 2.3.1. FT-IR Measurement

FTIR spectra were recorded on a NEXUS NICOLET FTIR spectrophotometer (Thermo Fisher Scientific, Waltham, MA, USA) in a KBr disk in the range from 4000 cm^−1^ to 400 cm^−1^. The spectra were analyzed by OMNIC™ 9.15 software (Thermo Fisher Scientific, Waltham, MA, USA).

#### 2.3.2. Raman Measurement

Raman spectra were recorded using the Renishaw inVia™ confocal Raman system (Renishaw, Wotton-under-Edge, UK), a high-performance instrument that integrates a research-grade microscope with an advanced spectrometer. This system allows for measurements with a temperature-controlled stage (Linkam, Redhill, UK) in the range of −196 °C to 1500 °C. Spectra were acquired using an 830 nm laser at 75% power, with 1200 L/mm grating (514/780) and a Renishaw Centrus 3CMC21 detector (Renishaw, Wotton-under-Edge, UK). Each spectrum was collected with 10 accumulations and an exposure time of 10 s, covering the spectral range from 100 cm^−1^ to 3200 cm^−1^. Data analysis was performed using Wire 5.5 software (Renishaw, Wotton-under-Edge, UK).

#### 2.3.3. pH Measurement

pH measurements were performed using a Multifunction Computer Meter CX-731 (Elmetron, Zabrze, Poland) equipped with a glass electrode.

#### 2.3.4. Scanning Electron Microscopy (SEM), Brunauer–Emmett–Teller (BET), and X-Ray Powder Diffraction (XRD) Analyses

The synthesized polymer’s surface morphology was characterized through SEM analysis conducted with a VEGA3 TESCAN microscope (TESCAN, Brno, Czech Republic). The specific surface area and pore volume were measured by an ASAP 2020 instrument (Micromeritics, Norcross, GA, USA). X-ray powder diffraction analysis was conducted with a Rigaku MiniFlex 600 instrument (Rigaku, Tokyo, Japan).

### 2.4. Sorption Tests

The sorption of methylene blue (MB) was investigated as a function of various process parameters, including the initial MB concentration, contact time, sorbent dosage, and the pH of the aqueous solution. A static method (batch tests) was employed for the experiments. Reactors with a volume of 25 cm^3^, containing varying polymer dosages (0.01–0.05 g), were supplemented with 10 cm^3^ of MB solution at different initial concentrations (10–100 mg/dm^3^). The pH of the aqueous solution (ranging from 2 to 12) was adjusted (prior to adding the solution to the polymer) using 0.1 M HCl or 0.1 M NaOH. The samples were shaken at a speed of 150 rpm for specific durations (1–90 min) at a temperature of 25 °C. Afterward, they were filtered, and the methylene blue content in the filtrate was determined. For shaking, a KS 4000 IC Control IKA incubator shaker was used, IKA Poland Ltd., Warszawa, Poland. The concentration of MB in the filtrate was determined using a UV/Vis UNICAM spectrophotometer at a wavelength of λ = 664 nm, Pye Unicam, Cambridge, UK.

The efficiency of MB removal from the model aqueous solution (*E*, %) was calculated using the following formula:

$$E=\left((c_{0}-c_{i}\right)/c_{0})\times 100\%$$

where

*c*_0_ is the initial MB concentration [mg/dm^3^].

*c_i_* is the final MB concentration [mg/dm^3^].

The sorption capacity of the polymer (*q_e_*, mg/g) was determined using the following formula:

$$q_{e}=\left((c_{0}-c_{i})\times V\right)/m$$

where

*V* is the volume of the solution (dm^3^).

*m* is the mass of the polymer (g).

### 2.5. Sorption Isotherms and Kinetics

In the adsorption process, the distribution of the adsorbate between the solution and the adsorbent is a crucial aspect. In the state of dynamic equilibrium, this distribution is precisely defined and described using adsorption isotherms. These isotherms illustrate the relationship between the quantity of adsorbed adsorbate, expressed as the amount per unit mass of the adsorbent, and the equilibrium concentration of the adsorbate in the solution at a constant temperature [26]. The most commonly used models to describe the sorption process include the Langmuir, Freundlich, and Temkin isotherms. These models take the form of mathematical–physical equations, enabling the description of phenomena occurring on the adsorbent surface.

The Langmuir isotherm is applied to quantify adsorption under the assumption that the adsorbent surface contains a specific number of active sites, proportional to the total surface area of the adsorbent. It is further assumed that only one molecule or one atom of adsorbate can be bound to each active site. Additionally, the adsorption centers on the surface of the solid are considered homogeneous in terms of energy, and adsorbed molecules remain non-interacting [17,26].

The Freundlich isotherm describes the sorption process based on the assumption of the distribution of active sites characterizing distinct surfaces of the adsorbent. Sorption proceeds in such a way that the number of molecules adsorbed at the full surface coverage of the adsorbent does not exceed the number of available active sites. The resulting layer on the adsorbent surface effectively isolates the action of adsorption forces, preventing the formation of any additional layers. The Freundlich model is particularly useful for describing multilayer sorption processes on heterogeneous surfaces and under variable concentrations of the adsorbate in the solution [17,26].

The Temkin isotherm describes a linear decrease in adsorption, which results from the uniform distribution of binding energy on the surface of the adsorbent. This model assumes that, during the sorption process, the interaction energy between the adsorbate and the adsorbent decreases linearly as the surface coverage increases. Consequently, the Temkin isotherm is particularly useful in the analysis of sorption processes where adsorption interactions are strongly dependent on the degree of surface saturation of the adsorbent [17,26].

The sorption kinetics were described using the pseudo-first-order and pseudo-second-order kinetic models; these models were expressed using equations from Lagiewka et al. (2023) [27].

## 3. Results and Discussion

### 3.1. Citric Acid–Linked β-Cyclodextrin Polymer

**Figure 1:**
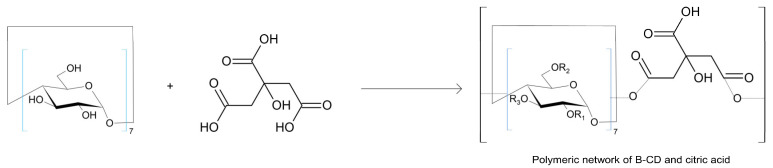
The synthesis route of the β-CD crosslinked polymer.

The β-cyclodextrin polymer crosslinked with citric acid at a weight ratio of 1:3 [CD:CA] assumes the form of an “ester,” where citric acid connects with β-cyclodextrin through a carbonyl bond: R-(C=O)-O-R′ [20]. The schematic mechanism of the formation of the beta-cyclodextrin polymer crosslinked with citric acid is presented in Figure 1.

### 3.2. The Characterization of the β-CD Polymer

**Figure 2:**
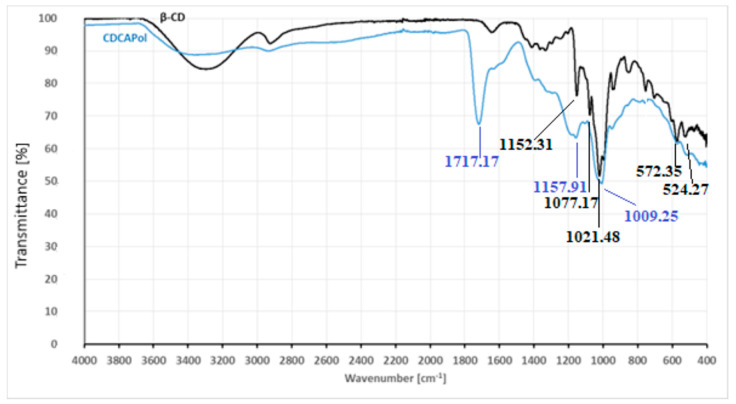
The FT-IR spectra of β-CD and CDCAPol.

The FT-IR spectra of β-CD and the β-cyclodextrin (β-CD) polymer are presented in Figure 2.

During the analysis of the FT-IR spectrum of the β-cyclodextrin (β-CD) polymer, special attention was given to the region characteristic of O-H group vibrations. In native β-CD, a broad band in the range of 3300–3500 cm^−1^ is typically observed, corresponding to the stretching vibrations of hydroxyl groups. In the case of the obtained β-CD polymer crosslinked with citric acid, this band is present and appears at approximately 3422 cm^−1^. The high intensity of this band also results from hydrogen bonds caused by interactions between the hydroxyl groups of β-CD and various carbonyl groups. The shift in this band indicates that an appropriate esterification reaction occurred between the –OH groups of β-CD and the carboxyl groups (C=O-OH) of citric acid, leading to the formation of the polymer structure. Another significant feature of the β-CD spectrum is the presence of a band at 2930 cm^−1^, corresponding to the stretching vibrations of C-H in alkyl groups. The presence of this band indicates that aliphatic structures remain present in the polymer network. A sharp and intense peak at 1734 cm^−1^ and a less intense band at 1654 cm^−1^ are associated with the stretching vibrations of carbonyl groups (C=O). One of these bands likely originates from ester fragments in the polymer network, while the other can be attributed to ketone groups present in diaryl structures. The band at 1636 cm^−1^, although of low intensity, corresponds to the vibrations of carboxylate groups (COO^−^), but its detection is challenging. Other significant bands were observed at 1209 cm^−1^, 1156 cm^−1^, and 1015 cm^−1^. These correspond to the stretching vibrations of C-O-C, C-C, and C-O-H bonds, resulting from characteristic vibrations in the glucose units of β-CD. The remaining bands present in the ranges of approximately 1700–1200 cm^−1^ and 760–530 cm^−1^ regions result from complex overlapping vibrational modes, including ring deformations, C-H bending, and skeletal vibrations of the β-CD polymer and ester network.

**Figure 3:**
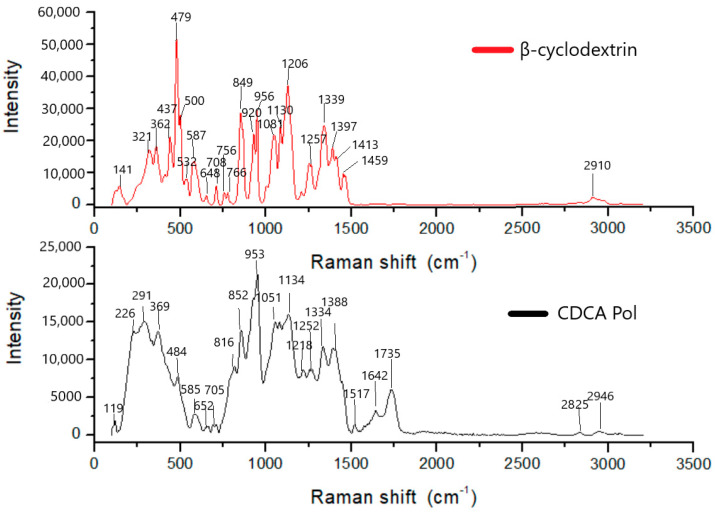
The Raman spectra of β-CD and CDCAPol.

The Raman spectra of β-CD and CDCAPol are presented in Figure 3.

The Raman spectrum of pure β-cyclodextrin (β-CD), shown in the upper graph in red, is characterized by numerous intense bands in the range of approximately 100 to 1500 cm^−1^, which correspond to typical structural vibrations of this molecule. The most prominent band, recorded at a Raman shift of 479 cm^−1^, is attributed to skeletal deformations of the glucose ring and C–O–C bond vibrations. Such a band is characteristic of the glucopyranose ring structure of sugars. In the range of approximately 849 to 956 cm^−1^, distinct signals appear that correspond to glycosidic bridges (C–O–C), which link the individual glucose units in the cyclodextrin structure. These bands are essential for confirming the integrity of the β-CD rings. The region between 1130 and 1257 cm^−1^, containing several overlapping signals (notably at 1130, 1206, and 1257 cm^−1^), relates to the stretching vibrations of C–C and C–O bonds within the molecule. In the higher range, between 1397 and 1459 cm^−1^, bands characteristic of CH_2_ and CH group deformations are observed. High intensity in this region is typical of sugars and their derivatives. Furthermore, at the end of the spectrum, in the region of 2910 cm^−1^, a weaker band was recorded, which can be attributed to the stretching vibrations of C–H bonds present in the methyl and methylene groups of the β-CD molecule. All identified bands are consistent with literature data for pure, unmodified β-cyclodextrin. Their presence confirms the absence of significant chemical alterations in the analyzed sample. The lower graph presents the Raman spectrum of a β-cyclodextrin sample that has been modified through crosslinking with citric acid. The observed spectral changes indicate structural transformations resulting from chemical reactions between the hydroxyl groups of β-CD and the carboxyl groups of citric acid. One of the most significant differences between the spectra is the appearance of a strong band at 1735 cm^−1^, which is absent in the spectrum of pure β-CD. This band is characteristic of carbonyl (C=O) stretching vibrations and indicates the presence of ester groups formed between the hydroxyl groups of β-CD and the carboxyl groups of citric acid. This is direct evidence of esterification and the formation of ester bridges within the polymer structure. Additional new signals are also observed in the higher Raman shift region—at 2825 and 2946 cm^−1^. Their presence indicates the stretching vibrations of CH_2_ and CH_3_ groups, which do not appear in such form in pure β-CD. This suggests the presence of chemically bound citric acid residues in the crosslinked network. It is also worth noting the changes in the 800–1300 cm^−1^ region. Although these bands are still present, their intensities and positions have shifted slightly. This may result from reduced rotational and vibrational freedom due to crosslinking, which affects the local chemical environment of the atoms. There is also a weakening of bands typical for intact glycosidic bridges, such as in the regions of 849, 1130, and 1206 cm^−1^, which may indicate partial chemical interference with the β-CD structure during the modification process. A comparative analysis of the Raman spectra clearly shows that the modification of β-cyclodextrin with citric acid leads to the formation of a crosslinked structure containing new ester groups, as confirmed by the band at 1735 cm^−1^. At the same time, the presence of characteristic cyclodextrin bands indicates that the core ring structure of the molecule has been partially preserved. The resulting polymeric material differs both chemically and spectrally from the original compound, confirming the effectiveness of the performed modification. In conclusion, Raman spectroscopy provides detailed insight into the chemical structure of the β-cyclodextrin–citric acid polymer. The presence of ester and carbonyl bands, along with preserved glycosidic and ring structures, confirms successful crosslinking and the formation of a three-dimensional polymeric network. Additionally, spectral changes reflect both covalent modifications and non-covalent interactions, such as hydrogen bonding, contributing to the stability and functionality of the resulting material.

**Figure 4:**
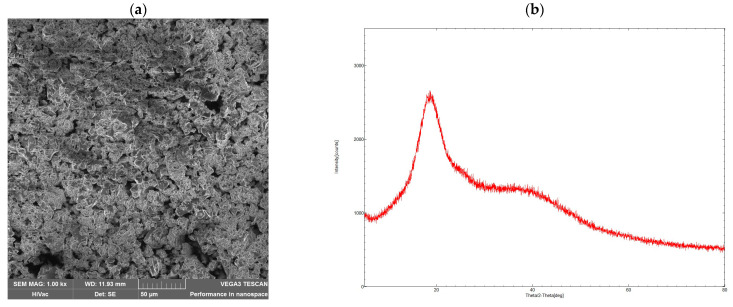
SEM image (**a**) and XRD pattern of CDCAPol (**b**).

The porosity characteristics of the polymer showed that CDCAPol possesses a low BET surface area of 0.81 m^2^/g. Calculations based on Barrett–Joyner–Halenda (BJH) theory applied to the N_2_ adsorption isotherms indicated a small pore volume of 1.61 × 10^−3^ cm^3^/g. Additionally, the SEM image (Figure 4a) confirmed the absence of significant porosity on the material’s surface. The XRD patterns shown in Figure 4b display the diffraction profiles of the β-cyclodextrin–citric acid polymer. The polymer’s pattern exhibits broad halo regions, indicating its amorphous nature and lack of crystallinity.

### 3.3. Sorption Process Depending on Variable Process Parameters

**Figure 5:**
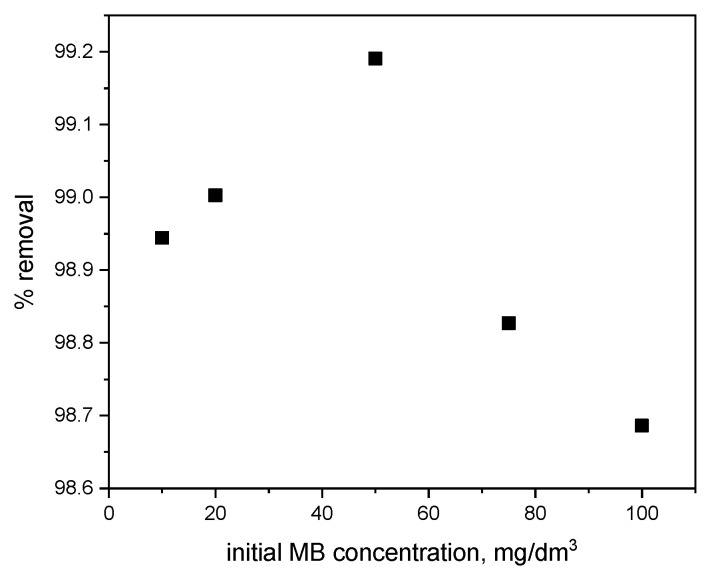
Effect of initial methylene blue concentration on its removal efficiency from aqueous solution (pH = 6; contact time = 60 min; adsorbent dose = 0.01 g).

The effect of the initial concentration of methylene blue (MB) on the efficiency of its removal from aqueous solution using the investigated polymer is presented in Figure 5.

Based on Figure 5, it was found that the highest percentage of methylene blue (MB) removal (99.2%) was achieved at an initial concentration of 50 mg/dm^3^. However, the removal efficiency for the remaining concentrations was also high, exceeding 98.6%. The overall efficiency of MB removal is very high (approximately 99–100%), indicating the effective performance of the polymer. At very high MB concentrations, slight decreases in efficiency may occur.

**Figure 6:**
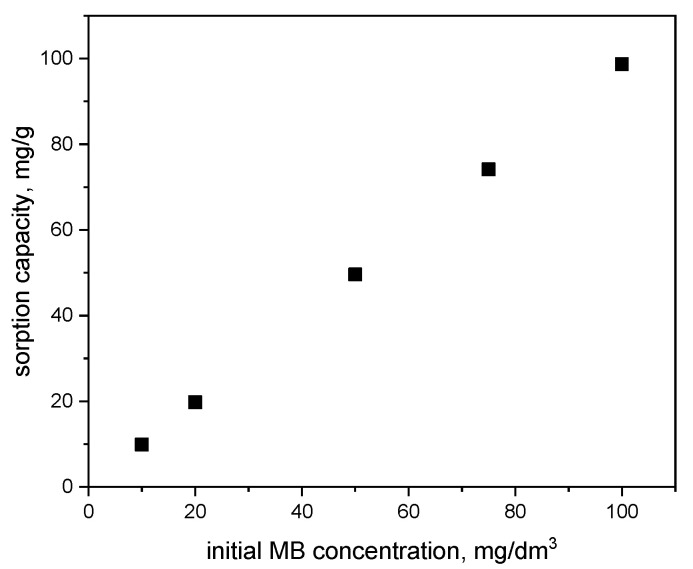
The uptake of MB of the polymer as a function of the initial methylene blue concentration (pH = 6; contact time = 60 min; adsorbent dose = 0.01 g).

The sorption capacity of the β-cyclodextrin-based polymer towards MB, depending on its initial concentration in water, is illustrated in Figure 6.

It was observed that the uptake of MB by the polymer increased with increasing dye concentration in water within the range of 10–100 mg/dm^3^. At an initial MB concentration of 100 mg/dm^3^, the MB uptake of the polymer reached 98.7 mg/g.

**Figure 7:**
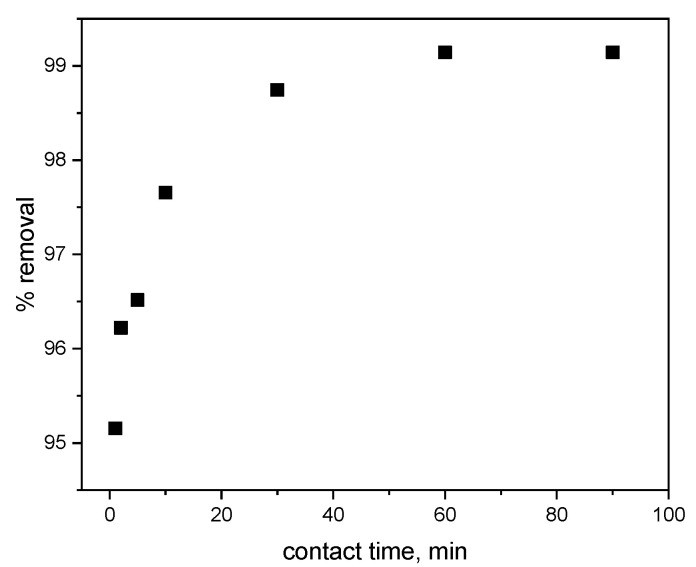
The effect of contact time on the efficiency of methylene blue removal from aqueous solution (pH = 6; adsorbent dose = 0.01 g; c_MB_ = 50 mg/dm^3^).

The effect of contact time on the efficiency of methylene blue removal from aqueous solution using the cyclodextrin-based polymer is presented in Figure 7.

The graph illustrates the effect of contact time on the efficiency of methylene blue (MB) removal from aqueous solution using a cyclodextrin-based polymer. The results presented in Figure 7 indicate that the percentage of MB removal increases with longer contact time. Notably, a contact time as short as 1 min resulted in over 95% removal, demonstrating the rapid and effective performance of the polymer in removing MB from aqueous solutions. At the initial stage of the process (up to approximately 30 min), a rapid increase in the removal percentage is observed, suggesting that the initial adsorption phase is highly dynamic and occurs at a fast rate. After about 60 min, the adsorption process reaches equilibrium. Prolonging the contact time beyond one hour does not further enhance the sorption efficiency or the extent of MB removal. The attainment of equilibrium suggests that most of the available adsorption sites on the polymer surface have been occupied.

The removal efficiency exceeds 99% after approximately 60 min and remains stable thereafter. These findings indicate that the optimal contact time is around 60 min, after which the adsorption process stabilizes. Moreover, the polymer exhibits a very high capacity for MB removal from aqueous solutions (over 99%), suggesting its potential effectiveness in practical water purification applications.

**Figure 8:**
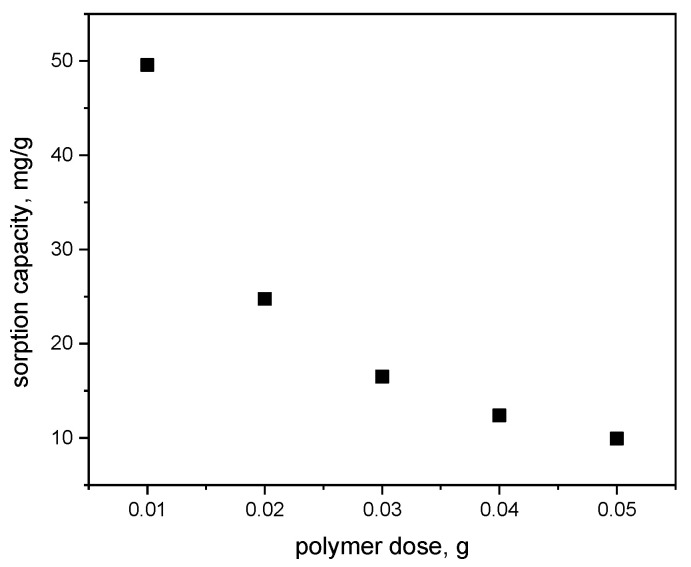
The sorption capacity of the polymer as a function of polymer dose (pH = 6; contact time = 60 min; c_MB_ = 50 mg/dm^3^).

The relationship between sorption capacity and polymer dose (q_e_ vs. polymer dose) indicates that the maximum sorption capacity (q_e_ ≈ 50 mg/g) is observed at the lowest polymer dose of 0.01 g (Figure 8). As the polymer dose increases, the sorption capacity gradually decreases, reaching approximately 10 mg/g at a dose of 0.05 g. The curve demonstrates a negative correlation between the adsorbent dose and sorption capacity, meaning that higher adsorbent doses result in a lower amount of MB adsorbed per unit mass of the polymer. This phenomenon may be attributed to particle agglomeration at higher doses, which reduces the surface area available for adsorption and limits the effectiveness of MB removal.

**Figure 9:**
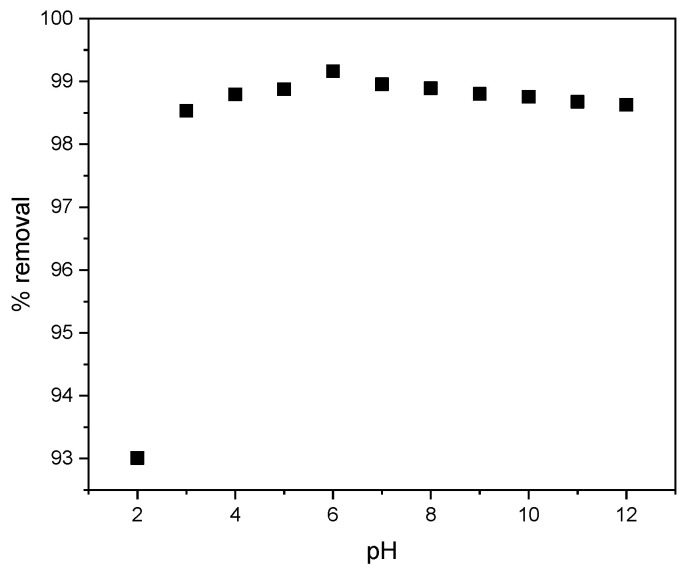
The effect of solution pH on the efficiency of methylene blue removal using the cyclodextrin-based polymer (contact time = 60 min; c_MB_ = 50 mg/dm^3^; dose = 0.01 g).

Figure 9 shows the effect of solution pH on the efficiency of methylene blue removal using the cyclodextrin-based polymer. The effectiveness of methylene blue (MB) removal remains very high across the entire pH range, fluctuating between approximately 93% and 100%. The highest removal efficiency (close to 100%) is observed in the pH range of 4–8, suggesting that the cyclodextrin-based polymer exhibits optimal adsorption performance in mildly acidic to neutral conditions. At low pH (pH = 2), the efficiency slightly decreases (~93%), likely due to the competition between H^+^ ions and MB for adsorption sites. At high pH (pH = 12), a slight decline in efficiency (~98%) is observed, which may result from changes in the surface charge of the polymer that hinder the adsorption of the cationic MB.

Based on the data, the adsorption mechanism can be interpreted as pH-dependent. At low (acidic) pH values, the positively charged surface of the adsorbent may repel the cationic methylene blue (MB), leading to reduced adsorption efficiency. In contrast, under neutral and alkaline conditions, the surface charge of the polymer becomes more negative, which favors electrostatic attraction with cationic MB, thereby enhancing removal efficiency.

Due to the high removal efficiency of MB across a wide pH range, the cyclodextrin-based polymer can be effectively applied under various environmental conditions without the need for precise pH control. This is particularly advantageous for practical applications such as wastewater treatment and water purification.

### 3.4. Sorption Behavior

**Figure 10:**
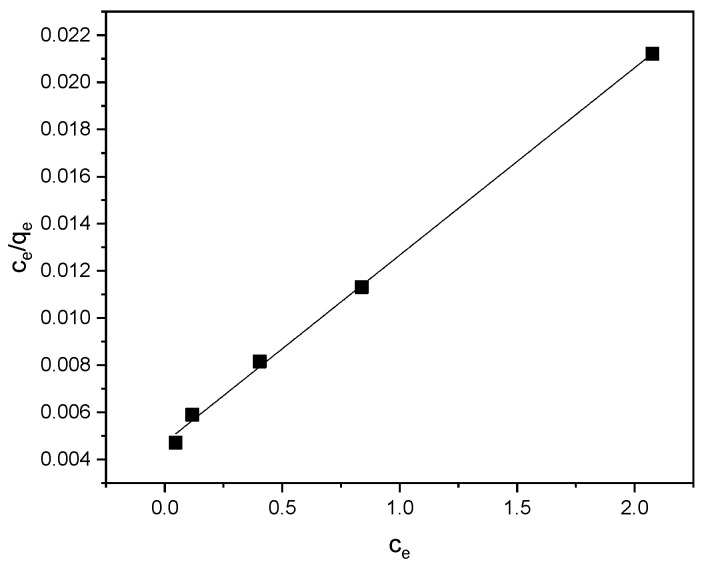
The Langmuir isotherm for the sorption of methylene blue onto the cyclodextrin-based polymer.

**Figure 11:**
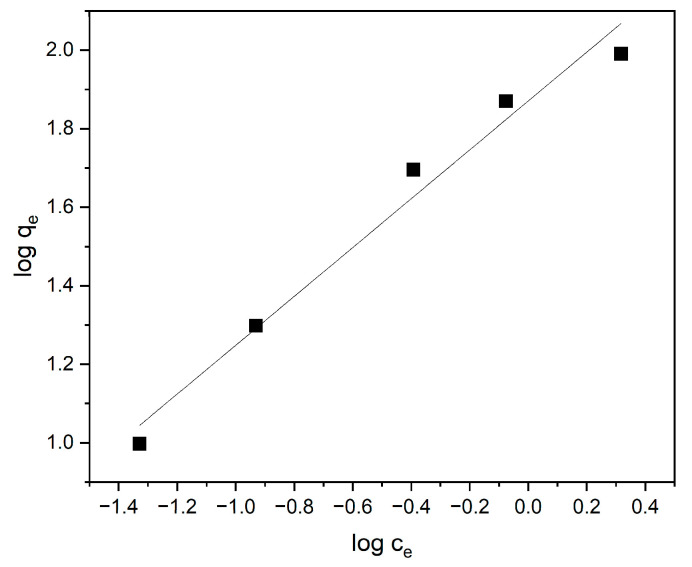
The Freundlich isotherm for the sorption of methylene blue onto the cyclodextrin-based polymer.

**Figure 12:**
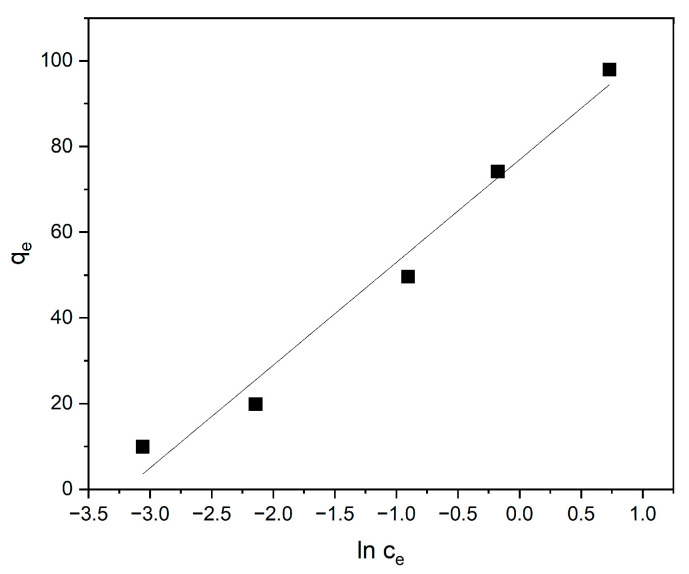
The Temkin isotherm for the sorption of methylene blue onto the cyclodextrin-based polymer.

The sorption isotherms corresponding to the Langmuir, Freundlich, and Temkin models are presented in Figure 10, Figure 11 and Figure 12, respectively. The parameters derived from these models are detailed in Table 1.

According to the Langmuir isotherm, adsorption takes place as a single layer on a surface with a fixed number of uniform sites. The fit of the experimental data to the Langmuir model suggests that the adsorption of methylene blue (MB) onto the cyclodextrin-based polymer proceeds via monolayer coverage, indicating homogeneous adsorption sites and uniform adsorption energies.

The Freundlich isotherm, shown in Figure 10, represents adsorption on heterogeneous surfaces and assumes a non-uniform distribution of adsorption heat and affinities. The shape of the Freundlich curve and the calculated parameters indicate favorable adsorption, particularly at low concentrations of MB, which may suggest the existence of various types of active sites on the polymer surface.

The Temkin isotherm assumes that the heat of adsorption of molecules in the layer decreases linearly with coverage due to adsorbate–adsorbent interactions. As shown in Figure 11, the Temkin model provides additional insight into the interaction energies between methylene blue and the polymer surface. The good fit of the experimental data to the Temkin isotherm suggests that adsorption is influenced by indirect adsorbate–adsorbent interactions and that the heat of adsorption decreases progressively with increasing surface coverage.

The Langmuir constant, k = 1.68 L/mg, determines the adsorption energy. A higher value suggests a stronger binding of methylene blue (MB) to the adsorbent surface. The maximum sorption capacity, Vm = 126.58 mg/g, indicates the amount of MB that can be adsorbed by the adsorbent under saturation conditions. The coefficient of determination, R^2^ = 0.9985, is very close to 1, indicating an excellent fit of the Langmuir model to the experimental data. The adsorption of MB best fits the Langmuir model, suggesting that a uniform monolayer has formed on the surface of the adsorbent.

The Freundlich constant, n = 1.61, indicates favorable adsorption. Since 1 < n < 2, it suggests that the adsorption is beneficial but not perfectly monolayer. The Freundlich model suggests adsorption on heterogeneous surfaces, which may indicate a variety of binding sites on the adsorbent.

The adsorption energy constant from the Temkin model, b = 0.10, indicates a physical adsorption process (since b < 1). The Temkin model indicates the physical nature of MB adsorption, meaning that MB adsorption is controlled by physical interactions, rather than chemical ones.

Based on the obtained results, the maximum sorption capacity of the β-cyclodextrin-based polymer crosslinked with citric acid for MB was determined to be 126.58 mg/g.

Various materials based on cyclodextrins, especially β-CD, have been used for the removal of methylene blue (MB) from aqueous solutions.

Liu et al. (2014) applied β-CD and poly(acrylic acid) nanocomposites, grafted onto graphene oxide [β-CD/PAA/GO], synthesized via esterification reactions [19]. These nanocomposites were used for the removal of cationic dyes, including methylene blue (MB) and safranine T, from aqueous solutions. Adsorption isotherms were fitted to the Langmuir model. The maximum adsorption capacity for MB removal from aqueous solutions was 247.99 mg/g. This excellent result was attributed to the high concentration of functional groups in the nanocomposites. The nanocomposites could be reused for multiple cycles of adsorption and desorption without a significant loss in adsorption capacity, even after five cycles.

In the work of Jiang et al., a crosslinked porous polymer based on β-CD containing citric acid groups (CT-β-CD) was synthesized [28]. The maximum sorption capacity for this adsorbent was 672 mg/g when applied for the removal of MB from aqueous solutions.

In 2008, Zhao and colleagues developed a β-CD polymer crosslinked with citric acid through polycondensation, which was insoluble in water [29]. The maximum sorption capacity (q_max_) for MB in this case was 105 mg/g.

In 2015, Zhao and his team produced a new water-insoluble composite adsorbent: sericin nanofibers/β-CD/poly(vinyl alcohol) [30]. This adsorbent was created through electrospinning and thermal crosslinking and was used for the removal of MB from aqueous solutions. The maximum adsorption capacity for MB removal was found to be 187.97 mg/g at a temperature of 293 K.

In 2021, Wang and colleagues developed a hydrogel through the crosslinking of β-CD and a functional monomer: acrylamide and 2-acrylamidomethylpropane-2-sulfonic acid [31]. After alkaline hydrolysis, the amide and sulfonic groups of the hydrogel were converted into carboxyl and sulfonate groups, which enhanced the adsorption of cationic dye ions (MB). The maximum adsorption capacity for methylene blue reached 2638.22 mg/g under equilibrium conditions

Cyclodextrins generally possess many hydroxyl groups, which can react with the carboxyl groups of polycarboxylic acids, such as citric acid. Due to this property, polymerization can occur, resulting in the in situ formation of a crosslinked polymer in a fibrous network [32]. On one hand, such polymers exhibit the inherent properties of cyclodextrins, including the ability to complex with other molecules. On the other hand, because esterification reactions are typically not complete, many free carboxyl groups remain on the polymer surface, leading to ion-exchange properties [32].

Each component of the polymer plays an important role in its adsorption function. The carboxyl groups not only act as linkers but also serve as adsorption sites for methylene blue. The cavities within the cyclodextrin rings contribute to trapping non-polar organic contaminants, which are also present in methylene blue—specifically the phenyl group and the azo cation. Given that surface binding sites are more accessible to the adsorbate than the cavity sites, the adsorption of methylene blue occurred on the surface of the β-cyclodextrin polymer through the carboxyl groups of citric acid [20].

In this study, the citric acid-crosslinked β-cyclodextrin polymer (CDCAPol) achieved a maximum adsorption capacity of 126.58 mg/g and a removal efficiency of 99.2% for methylene blue (MB) under optimized conditions. These results are promising but moderate compared to the values reported in the literature. For example, Liu et al. (2014) reported a capacity of 247.99 mg/g for a β-CD/poly(acrylic acid)/graphene oxide composite [19], while Wang et al. (2021) achieved an exceptionally high capacity of 2638.22 mg/g using a sulfonated β-CD-based hydrogel [31]. Jiang et al. (2019) obtained 672 mg/g with a citric acid-containing porous β-CD polymer [28], and Zhao et al. (2015) reported 187.97 mg/g using a β-CD/PVA/sericin composite [30]. Although the CDCAPol polymer does not reach these maximum values, it benefits from a simpler, environmentally friendly synthesis using biodegradable components and mild thermal crosslinking, which may offer advantages in terms of cost, scalability, and sustainability. Thus, while the adsorption capacity is moderate, the material’s ease of preparation and high removal efficiency still support its potential for practical water treatment applications.

Beyond β-cyclodextrin-based sorbents, a wide range of polymeric adsorbents have been developed and tested for dye removal. For example, polyaniline-based hydrogels have shown high adsorption capacities for methylene blue, reaching over 500 mg/g, due to their extended π-conjugation and strong electrostatic interactions with cationic dyes [33]. Similarly, chitosan-based composites modified with graphene oxide or magnetic nanoparticles achieve capacities in the range of 200–600 mg/g, depending on pH, crosslinking degree, and surface area [34]. Synthetic polymers such as polyacrylamide and sulfonated polystyrene have also demonstrated good affinity for dyes, though they often require further functionalization to enhance selectivity and efficiency [35]. Compared to these materials, the cyclodextrin-based CDCAPol developed in this study offers competitive efficiency while maintaining eco-friendly synthesis routes, biodegradability, and selective host–guest complexation, making it a promising alternative for practical wastewater treatment applications.

**Figure 13:**
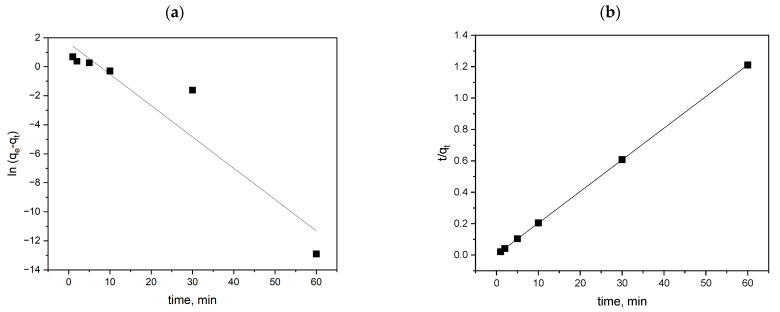
Pseudo-first- (**a**) and pseudo-second-order (**b**) kinetics for the removal of MB on the cyclodextrin polymer (pH = 6; dose = 0.01 g; c_MB_ = 50 mg/dm^3^).

The pseudo-first- and pseudo-second-order kinetics are presented in Figure 13a and Figure 13b, respectively, and the model parameters are listed in Table 2.

The adsorption rate constant k_1_ = 0.22 L/min suggests a moderately fast adsorption process. The calculated value of q_e_ = 5.17 mg/g significantly deviates from the experimental value 49.58 mg/g, indicating that the first-order model does not accurately predict the actual adsorption capacity. The coefficient R^2^ = 0.8977 suggests a reasonable, but not ideal, agreement between the model and the experimental data. The pseudo-first-order model does not fit the data well, which means that the adsorption of MB on the cyclodextrin polymer is not a process governed by internal diffusion as the rate-limiting step.

The adsorption rate constant k_2_ = 0.18 g/mg⋅min is relatively high, indicating an intensive adsorption process. The calculated value of q_e_ = 49.75 mg/g is almost identical to the experimental value 49.58 mg/g, indicating a very good agreement between the model and the experimental data. The determination coefficient R^2^ = 0.999 shows the excellent fit of the pseudo-second-order kinetic model to the experimental data.

The results fit the pseudo-second-order model and suggest that MB removal by the cyclodextrin polymer involves interactions between the sorbent functional groups and the dye molecules. The adsorption of MB on the cyclodextrin polymer results from the interaction of the sorbate particles with the carboxyl groups of citric acid.

## 4. Conclusions

Based on the obtained results, it was concluded that the citric acid-crosslinked beta-cyclodextrin polymer is an effective “agent” for removing the organic contaminant, methylene blue, from water. The highest methylene blue removal efficiency (99.2%) was achieved for the following process parameters: polymer dose = 0.01 g, corresponding to a concentration of 1 g/dm^3^, pH = 6, and an initial MB concentration of 50 mg/dm^3^. The maximum sorption capacity of the polymer for MB was 126.58 mg/g.

The reduction in MB concentration in aqueous solutions through the use of the cyclodextrin polymer is based on the physical adsorption of ions on the surface of this material via binding to the carboxyl groups of citric acid.

The cyclodextrin-based polymer’s high MB removal efficiency across a wide pH range allows for effective use under varying environmental conditions without precise pH control, making it highly suitable for applications such as wastewater treatment and water purification.

While the citric acid-crosslinked β-cyclodextrin polymer shows considerable potential as an efficient sorbent for water treatment, future studies should address its performance and competitive uptake behavior in the presence of diverse organic and inorganic contaminants in real environmental matrices.

## Figures and Tables

**Table 1:** Sorption isotherm constants for the Langmuir, Freundlich, and Temkin models.

	Langmuir Isotherm			Freundlich Isotherm			Temkin Isotherm		
	k	V_m_	R^2^	K_f_	n	R^2^	K_T_	b	R^2^
MB	1.68	126.58	0.9985	74.22	1.61	0.9779	24.73	0.10	0.9780

**Table 2:** The model parameters of pseudo-first-order and pseudo-second-order kinetics for the removal of MB on the cyclodextrin polymer.

	q_e dośw_ mg/g	Pseudo-First-Order Kinetic Equation			Pseudo-Second-Order Kinetic Equation		
		k_1_ L/min	q_e obl_ mg/g	R^2^	k_2_ g/mg min	q_e obl_ mg/g	R^2^
MB	49.58	0.22	5.17	0.8977	0.18	49.75	0.999

## Data Availability

The original contributions presented in this study are included in the article. Further inquiries can be directed to the corresponding author.
